# Associations between MTHFR gene polymorphisms (C677T and A1298C) and genetic susceptibility to prostate cancer: a systematic review and meta-analysis

**DOI:** 10.3389/fgene.2024.1343687

**Published:** 2024-01-26

**Authors:** Jianan You, Yuhua Huang, Xinyu Shen, Yunyi Chen, Xiang Ding

**Affiliations:** Department of Urology, First Affiliated Hospital of Soochow University, Suzhou, Jiangsu, China

**Keywords:** MTHFR, C677T, A1298C, genetic polymorphism, prostate cancer, rs1801133, rs1801131

## Abstract

**Background:** The association between MTHFR gene polymorphisms (C677T and A1298C) and prostate cancer risk remains controversial.

**Methods:** Two independent researchers searched the PubMed, Embase, Cochrane and Web of Science databases for all papers published up to 12/19/2023 and used various genetic models to evaluate the relationship between MTHFR polymorphisms and prostate cancer risk.

**Results:** The meta-analysis included 26 case‒control studies with a total of 12,455 cases and 13,900 controls with the C677T polymorphism and 6,396 cases and 8,913 controls with the A1298C polymorphism. Overall, no significant association was found between the MTHFR gene polymorphisms and prostate cancer risk. However, the C677T polymorphism was associated with reduced prostate cancer risk in the Asian population (T allele vs. C allele: OR = 0.759, 95% CI 0.669–0.861, *p* < 0.001; TT + CT vs. CC: OR = 0.720, 95% CI 0.638–0.812, *p* < 0.001; TT vs. CC + CT: OR = 0.719, 95% CI 0.617–0.838, *p* < 0.001; TT vs. CC: OR = 0.620, 95% CI 0.522–0.737, *p* < 0.001); however, the A1298C polymorphism was associated with an increased risk in the mixed race group from the United States (CC + AC vs. AA: OR = 1.464, 95% CI 1.052–2.037, *p* = 0.024; AC vs. AA: OR = 1.615, 95% CI 1.037–2.514, *p* = 0.034).

**Conclusion:** The meta-analysis suggested that MTHFR gene polymorphisms (C677T and A1298C) may have different effects on prostate cancer risk in specific populations.

## 1 Introduction

Prostate cancer is a common malignant disease in males that originates from cancer cells within prostate tissue; prostate tumours often grow slowly and remain asymptomatic (Ferlay et al., 2019). According to data from the World Health Organization (WHO), prostate cancer ranks second in incidence among males worldwide, second only to lung cancer (Ferlay et al., 2021). The incidence of prostate cancer is greater among older men, especially those aged >60 years (Siegel et al., 2022). Prostate cancer is also associated with higher socioeconomic status (Chen et al., 2018). In China, the incidence of prostate cancer has been increasing, with an annual incidence rate of 39.09 cases per 100,000 persons and a 5-year survival rate of 79.8% according to the 2018 China Cancer Registry Annual Report (Descotes, 2019).

Prostate cancer is a multifactorial disease associated with risk factors, including age, family history, insulin-like growth factor levels, dietary habits, lifestyle factors, environmental factors, and occupational exposure (Perdana et al., 2016). A descriptive epidemiological study conducted by Pernar et al. emphasized that age, family history, race, and genetics were significant risk factors for prostate cancer (Malik et al., 2018). Hence, genetic polymorphisms play a crucial role in prostate cancer susceptibility. Methylenetetrahydrofolate reductase (MTHFR) is a crucial enzyme involved in DNA methylation. Mutations in the MTHFR gene may lead to changes in enzyme activity, resulting in alterations in DNA methylation levels and an increased risk of prostate cancer. A1298C and C677T are two common mutation sites in the MTHFR gene. The A1298C mutation may cause decreased enzyme activity, affecting the extent of DNA methylation and increasing the risk of prostate cancer. The C677T mutation, on the other hand, can affect enzyme stability and function, leading to disruption of the maintenance of DNA methylation levels and an increased risk of prostate cancer (Choi and Friso, 2006; Dong et al., 2008). Therefore, understanding the relationship between these two mutation sites and prostate cancer may provide new insights for early diagnosis and treatment.

However, the results of previous meta-analyses vary across studies. For example, some studies found no association between the A1298C or C677T locus and prostate cancer, while others showed a clear association (Collin et al., 2009; Li et al., 2012; Li and Xu, 2012; Zhang et al., 2012; Chen et al., 2015). In addition, previous meta-analyses did not perform subgroup analyses for different races, genetic testing methods, or sample types, which may also lead to differences in results. Although some studies have explored the relationship between the A1298C and C677T loci and prostate cancer, additional studies are needed to determine the importance of this association. To address this significant and controversial question, we performed an updated meta-analysis based on current clinical evidence to assess the association between MTHFR gene polymorphisms (C677T and A1298C) and prostate cancer risk.

## 2 Methods and materials

### 2.1 Search strategy

Two independent researchers searched the PubMed, Embase, Cochrane and Web of Science databases for all papers published up to 12/19/2023. The following keywords were used: “prostate tumour”, “prostatic neoplasms”, “prostate cancer”, “MTHFR”, “methylenetetrahydrofolate reductase”, “A1298C″, “rs1801131″, “Glu429Ala”, “C677T″, “rs1801133″, and “Ala222Val”. To ensure a comprehensive literature search, the recommended MeSH terms were used for each keyword with similar meanings. In addition, the reference lists of the included studies were manually searched to identify other published articles not indexed in public databases (Supplementary Appendix S1).

### 2.2 Data extraction

Two researchers extracted and crosschecked the data based on predefined inclusion and exclusion criteria. If the data were insufficient or uncertain, attempts were made to contact the original authors to supplement and verify the accuracy of the data. Incomplete studies were excluded, and only the highest-quality studies were retained among those with duplicate publications, replication, or similar data.

The following information was extracted: the surname of the first author, publication year, country, and participant race. Additionally, the number of cases and controls, matching variables, and the genotype distributions of the cases and controls from the data sources were recorded.

### 2.3 Quality assessment

On this quality assessment scale, six items were evaluated (Ferlay et al., 2019): appropriateness of the case definition and diagnosis (Ferlay et al., 2021), representativeness of cases (Siegel et al., 2022), selection of controls (Chen et al., 2018), definition of controls (Descotes, 2019), investigation and assessment of exposure, and (Perdana et al., 2016) similarity of investigation methods between cases and controls. The quality score ranged from 0 to 9. Studies scoring less than 4 points were considered low-quality studies, studies scoring 4–6 points were considered moderate-quality studies, and those scoring >6 pints were considered high-quality studies.

### 2.4 Data analysis

By calculating the combined odds ratios (ORs) and corresponding 95% confidence intervals (95% CIs) of the gene frequencies for each genetic model and considering *p* < 0.05 to indicate statistical significance, we evaluated the association between the MTHFR C677T and A1298C polymorphisms and the risk of prostate cancer. We compared five genetic models (Ferlay et al., 2019): an allelic model (C677T: T allele vs. C allele; A1298C: C allele vs. A allele) (Ferlay et al., 2021); a dominant model [C677T: (TT + CT) vs. CC; A1298C: (CC + AC) vs. AA] (Siegel et al., 2022); a recessive model (C677T: TT vs. (CC + CT); A1298C: CC vs. (AA + AC); (Chen et al., 2018) an overdominant model (C677T: CT vs. TT; A1298C: AC vs. CC); and (Descotes, 2019) an additive model (C677T: TT vs. CC; A1298C: CC vs. AA). We assessed heterogeneity among the included studies using the chi-square-based Q test and I^2^ test. A *p*-value < 0.10 indicated heterogeneity among the studies (Cumpston et al., 2019). If the I^2^ value was <40%, the heterogeneity was likely unimportant, whereas if the I^2^ value was >75%, it was believed that there was considerable heterogeneity (Chang et al., 2022). Due to unavoidable differences in heterogeneity, a random effects model (REM) was used for quantitative meta-analysis (Khandagale et al., 2023). Additionally, subgroup analyses were performed to investigate the potential sources of heterogeneity. Subgroup analyses were performed based on race, genotyping method, and sample type, and Hardy-Weinberg equilibrium (HWE) was calculated using the chi-square goodness-of-fit test, with *p* > 0.05 indicating HWE in the control group; otherwise, Hardy-Weinberg disequilibrium (HWD) was indicated. Sensitivity analysis was conducted by sequentially excluding each study, and if the sensitivity analysis results were not robust within an acceptable range, the study was removed for reanalysis. Begg’s funnel plot and Egger’s test were employed to assess the publication bias risk in the selected studies. All the abovementioned statistical analyses were performed using STATA 15.0.

## 3 Results

### 3.1 Study characteristics

**FIGURE 1:**
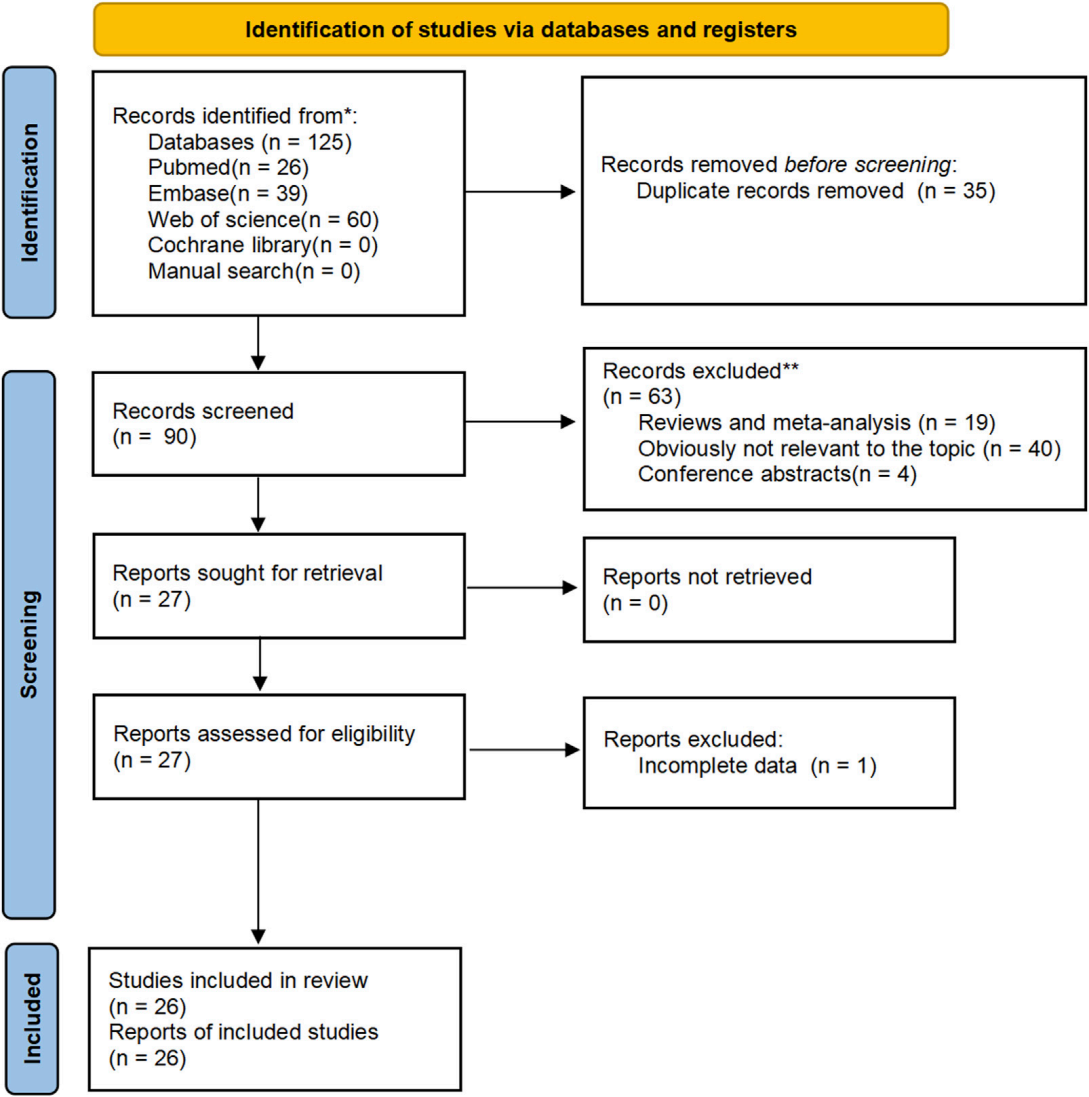
Flow chart of meta-analysis to identify associations between MTHFR gene polymorphisms (C677T, A1298C) and prostate cancer risk.

A literature search and screening of the Embase, PubMed, Web of Science, and Cochrane databases resulted in a total of 39 articles from Embase, 26 articles from PubMed, 60 articles from Web of Science, and no articles from the Cochrane database or manual search. The detailed screening process is illustrated in Figure 1. After reading the titles and abstracts, 99 articles were excluded; 35 were duplicates, 40 were irrelevant to the topic, 19 were reviews or meta-analyses, 4 were conference abstracts, and 1 had incomplete data. Overall, our meta-analysis included 26 eligible case‒control studies (Figure 1). Among the 26 studies, all studies investigated C677T, while 14 studies investigated A1298C. The genotype frequencies of C677T and A1298C were separately reported; thus, these reports were considered individual studies in this meta-analysis. Therefore, all 26 studies (Kimura et al., 2000; Heijmans et al., 2003; Cicek et al., 2004; Singal et al., 2004; Van Guelpen et al., 2006; Johansson et al., 2007; Reljic et al., 2007; Marchal et al., 2008; Stevens et al., 2008; Collin et al., 2009; Muslumanoglu et al., 2009; Cai et al., 2010; Safarinejad et al., 2010; Wu et al., 2010; Kosova et al., 2011; Küçükhüseyin et al., 2011; Fard-Esfahani et al., 2012; Kobayashi et al., 2012; Mandal et al., 2012; Vidal et al., 2012; de Vogel et al., 2013; López-Cortés et al., 2013; Ghasemi et al., 2014; Wu et al., 2016; TELLOUCHE-BOUHOUHOU et al., 2018; Mouhoub-Terrab et al., 2023) included 12,455 cases and 13,900 controls with the C677T polymorphism and 6,396 cases and 8,913 controls with the A1298C polymorphism (Table 1).

**TABLE 1:** Characteristics of studies included in the meta-analysis.

Study	Year	Country	Race	Sample size case/control	Study source	Character of control (matched for)	Sample source	Genotyping	P For HWE
C677T									
Kimura et al	2000	Germany	Caucasian	132/150	Hospital	Healthy	Peripheral blood	PCR-RFLP	0.169
Heijmans et al	2003	Nrtherland	Caucasian	21/772	Population	Healthy (age)	Peripheral blood	PCR-RFLP	0.690
Cicek et al	2004	United States	Mixed	439/479	Family	Sibling	Peripheral blood	PCR-RFLP	0.139
Singal et al	2004	United States	Mixed	81/42	Hospital	BPH	Paraffinized tissue	PCR-RFLP	0.280
Van Guelpen et al	2006	Sweden	Caucasian	223/435	Population	Healthy (age, sampling date)	Peripheral blood	TaqMan	0.128
Reljic et al	2007	Croatia	Caucasian	95/37	Hospital	Healthy (age)	Peripheral blood	PCR-RFLP	0.025
Johansson et al	2007	Sweden	Caucasian	2,677/1,541	Population	Healthy (age, county)	Peripheral blood	TaqMan	0.468
Marchal et al	2008	Spain	Caucasian	182/204	Hospital	BPH	Peripheral blood	TaqMan	0.022
Stevens et al	2008	United States	Mixed	1,100/1,107	Population	Healthy (age, sampling date, ethnicity)	Peripheral blood	TaqMan	0.983
Muslumanoglu et al	2009	Turkey	Caucasian	93/157	Hospital	BPH	Peripheral blood	PCR-RFLP	0.810
Collin et al	2009	United Kingdom	Caucasian	1,599/2084	Population	Healthy	Peripheral blood	TaqMan	0.259
Cai et al	2010	China	Asian	217/220	Hospital	BPH	Peripheral blood	PCR-RFLP	0.383
Wu et al	2010	Taiwan	Asian	218/436	Population	Healthy (age, habits)	Peripheral blood	PCR-RFLP	0.763
Safarinejad et al	2010	Iran	Caucasian	174/348	Population	Healthy (age)	Peripheral blood	PCR-RFLP	0.938
Kosova et al	2011	Turkey	Caucasian	112/145	Hospital	Healthy	Peripheral blood	PCR-RFLP	0.486
Kucukhuseyin et al	2011	Turkey	Caucasian	55/50	Hospital	Healthy (age)	Peripheral blood	PCR-RFLP	0.017
Mandal et al	2012	India	Caucasian	195/250	Population	Healthy (age, ethnicity)	Peripheral blood	PCR-RFLP	0.134
Kobayashi et al	2012	Canada	Caucasian	43/170	Hospital	Urology controls	Peripheral blood	PCR-RFLP	0.042
Frad-Esfahani et al	2012	Iran	Caucasian	67/75	Hospital	BPH	Paraffinized tissue	ARMS-PCR	0.071
Vidal et al	2012	United States	Caucasian	55/192	Hospital	Healthy	Peripheral blood	Sequenom MassArry	0.267
Lopez-Cortes et al	2013	Ecuador	Caucasian	104/110	Population	Healthy	Paraffinized tissue	PCR-RFLP	0.001
Vogel et al	2013	Norway	Caucasian	2,522/2,607	Population	Healthy (age, sampling date, county)	Peripheral blood	MLDMS	0.000
Ghasemi et al	2014	Iran	Caucasian	30/40	Hospital	Healthy (age)	Peripheral blood	ARMS-PCR	0.608
Wu et al	2016	Taiwan	Asian	1817/2026	Population	Healthy (age)	Peripheral blood	PCR-RFLP	0.412
Samah et al	2018	Algeria	Caucasian	98/98	Hospital	Healthy (age)	Peripheral blood	PCR-RFLP	0.025
Mouhoub-Terrab et al	2023	Algeria	Caucasian	106/125	Hospital	Healthy (age)	Peripheral blood	TaqMan	0.840
A1298C									
Cicek et al	2004	United States	Mixed	439/479	Family	Sibling	Peripheral blood	PCR-RFLP	0.945
Singal et al	2004	United States	Mixed	81/42	Hospital	BPH	Paraffinized tissue	PCR-RFLP	0.396
Van Guelpen et al	2006	Sweden	Caucasian	222/434	Population	Healthy (age, sampling date)	Peripheral blood	TaqMan	0.765
Marchal et al	2008	Spain	Caucasian	177/209	Hospital	BPH	Peripheral blood	TaqMan	0.194
Stevens et al	2008	United States	Mixed	1,104/1,109	Population	Healthy (age, sampling date, ethnicity)	Peripheral blood	TaqMan	0.941
Muslumanoglu et al	2009	Turkey	Caucasian	91/166	Hospital	BPH	Peripheral blood	PCR-RFLP	0.000
Collin et al	2009	United Kingdom	Caucasian	1,592/3,035	Population	Healthy	Peripheral blood	TaqMan	0.249
Cai et al	2010	China	Asian	217/220	Hospital	BPH	Peripheral blood	PCR-RFLP	0.270
Wu et al	2010	Taiwan	Asian	218/436	Population	Healthy (age, habits)	Peripheral blood	PCR-RFLP	0.697
Safarinejad et al	2010	Iran	Caucasian	174/348	Population	Healthy (age)	Peripheral blood	PCR-RFLP	0.628
Vidal et al	2012	United States	Caucasian	55/193	Hospital	Healthy	Peripheral blood	Sequenom MassArry	0.450
Lopez-Cortes et al	2013	Ecuador	Caucasian	104/110	Population	Healthy (age, sampling date, county)	Paraffinized tissue	PCR-RFLP	0.000
Wu et al	2016	Taiwan	Asian	1817/2026	Population	Healthy (age)	Peripheral blood	PCR-RFLP	0.519
Mouhoub-Terrab et al	2023	Algeria	Caucasian	105/106	Hospital	Healthy (age)	Peripheral blood	TaqMan	0.798

The participants included Caucasians, Asians, and individuals of mixed races. Genotyping of the included SNPs was performed using TaqMan, polymerase chain reaction-restriction fragment length polymorphism (PCR-RFLP), an amplification refractory mutation system (ARMS), Sequenom MassArray, and matrix-assisted laser desorption/ionization-time-of-flight mass spectrometry (MALDI-TOF MS). According to the methodological quality assessment, all studies had scores above 4. The genotype distributions of the control groups in all studies were tested for Hardy‒Weinberg equilibrium (HWE) (Table 1), and studies deviating from HWE were excluded from subsequent analyses. Thus, the final analysis included 19 studies for the C677T polymorphism, comprising 9,356 cases and 10,624 controls, and 12 studies for the A1298C polymorphism, comprising 6,201 cases and 8,637 controls.

### 3.2 Association between the C677T (rs1801133) polymorphism and prostate cancer susceptibility

**FIGURE 2:**
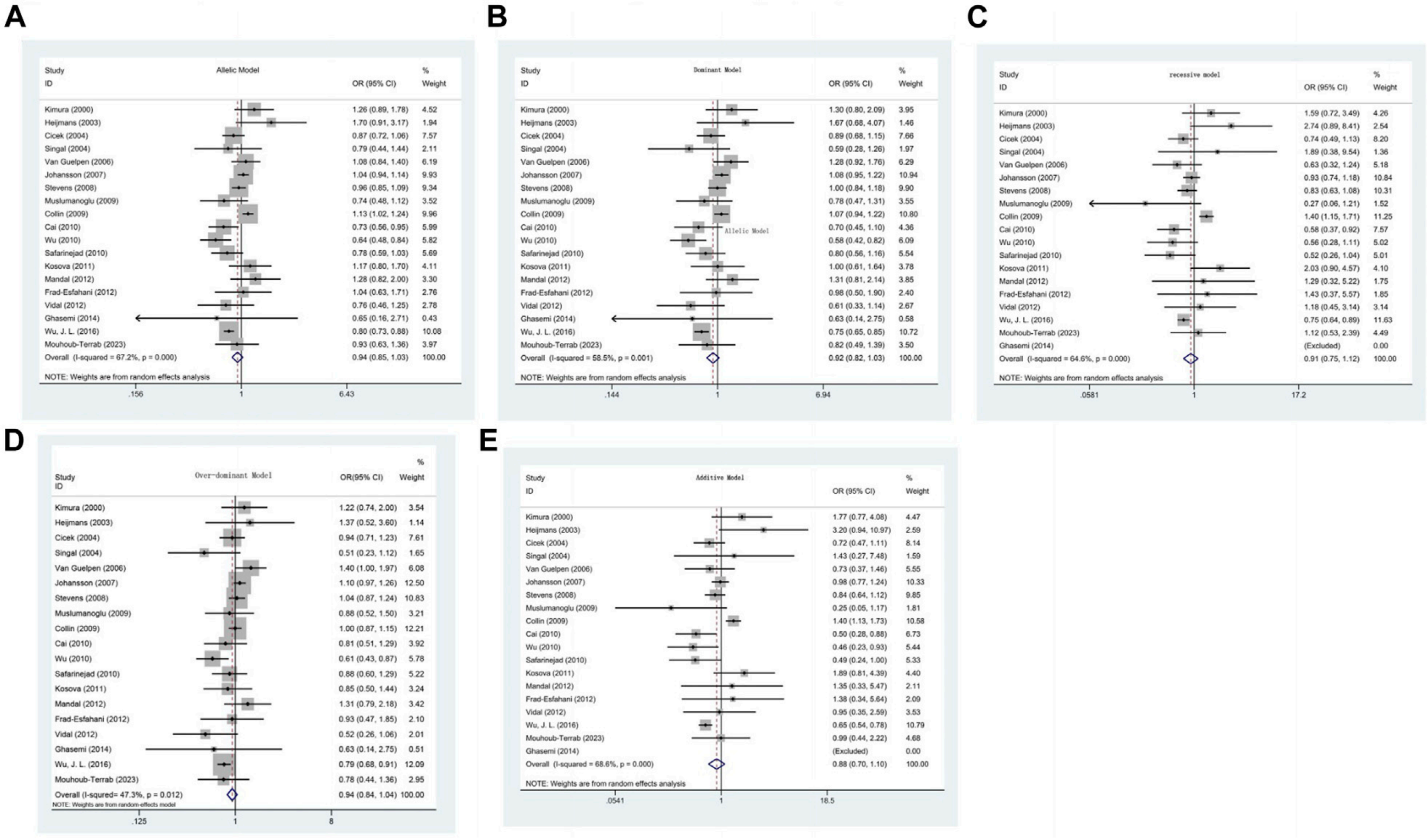
**(A)** Forest plot of the association between MTHFR gene C677T polymorphism and PCa risk under Allelic model. **(B)** Forest plot of the association between MTHFR gene C677T polymorphism and PCa risk under Dominant model. **(C)** Forest plot of the association between MTHFR gene C677T polymorphism and PCa risk under recessive model. **(D)** Forest plot of the association between MTHFR gene C677T polymorphism and PCa risk under over-dominant model. **(E)** Forest plot of the association between MTHFR gene C677T polymorphism and PCa risk under additive model.

The strength of the association between the C677T (rs1801133) polymorphism and prostate cancer risk is presented in Table 2. As shown in Table 2, no significant associations were detected in any of the genetic models (T allele vs. C allele: OR = 0.935, 95% CI 0.850–1.029, *p* = 0.171, I^2^ = 62.7%, *p*_H_ < 0.001; TT + CT vs. CC: OR = 0.920, 95% CI 0.820–1.032, *p* = 0.154, I^2^ = 58.5%, *p*_H_ = 0.001; TT vs. CC + CT: OR = 0.915, 95% CI 0.749–1.117, *p* = 0.381, I^2^ = 64.6%, *p*_H_ < 0.001; CT vs. CC: OR = 0.936, 95% CI 0.841–1.041, *p* = 0.225, I^2^ = 47.3%, *p*_H_ = 0.012; TT vs. CC: OR = 0.882, 95% CI 0.704–1.104, *p* = 0.272, I^2^ = 68.6%, *p*_H_ < 0.001) (Table 2) (Figures 2A–E).

**TABLE 2:** Meta-analysis between MTHFR C677T polymorphism and PCa risk under genetic models.

Classification	N	Contrasting model	OR	95%CI	P	Heterogeneity		Publication bias	
						P Of Q-test	I^2^%	P Of Begg test	P Of Egger test
Total	19	T *versus* C	0.935	0.850–1.029	0.171	0.000	67.2	0.780	0.775
		TT + CT *versus* CC	0.920	0.820–1.032	0.154	0.001	58.5	0.294	0.508
		TT *versus* CC + CT	0.915	0.749–1.117	0.381	0.001	64.6	0.289	0.837
		CT *versus* TT	0.936	0.841–1.041	0.225	0.012	47.3	0.234	0.382
		TT *versus* CC	0.882	0.704–1.104	0.272	0.000	68.6	0.289	0.910
Race									
Caucasian	13	T *versus* C	1.045	0.958–1.140	0.324	0.199	24.2		
		TT + CT *versus* CC	1.059	0.979–1.144	0.151	0.461	0.0		
		TT *versus* CC + CT	1.093	0.835–1.019	0.518	0.014	53.5		
		CT *versus* TT	1.041	0.952–1.138	0.381	0.407	4.0		
		TT *versus* CC	1.089	0.833–1.422	0.533	0.029	48.8		
Asian	3	T *versus* C	0.759	0.669–0.861	0.000	0.249	28.1		
		TT + CT *versus* CC	0.720	0.638–0.812	0.000	0.409	0.0		
		TT *versus* CC + CT	0.751	0.636–0.887	0.000	0.442	0.0		
		CT *versus* TT	0.761	0.670–0.865	0.000	0.416	0.0		
		TT *versus* CC	0.620	0.522–0.737	0.000	0.477	0.0		
Mixed	3	T *versus* C	0.928	0.837–1.029	0.157	0.635	0.0		
		TT + CT *versus* CC	0.943	0.815–1.091	0.433	0.352	4.1		
		TT *versus* CC + CT	0.816	0.653–1.019	0.073	0.537	0.0		
		CT *versus* TT	0.949	0.762–1.181	0.638	0.208	36.4		
		TT *versus* CC	0.815	0.645–1.030	0.087	0.668	0.0		
Genotyping method									
PCR-RFLP	11	T *versus* C	0.878	0.769–1.003	0.056	0.010	56.7		
		TT + CT *versus* CC	0.839	0.720–0.977	0.024	0.070	41.8		
		TT *versus* CC + CT	0.841	0.635–1.115	0.229	0.022	52.2		
		CT *versus* TT	0.848	0.746–0.965	0.012	0.266	18.7		
		TT *versus* CC	0.791	0.574–1.089	0.151	0.009	57.3		
TaqMan	5	T *versus* C	1.047	0.981–1.117	0.169	0.331	13.0		
		TT + CT *versus* CC	1.063	0.985–1.146	0.116	0.592	0.0		
		TT *versus* CC + CT	0.989	0.748–1.307	0.937	0.006	72.1		
		CT *versus* TT	1.061	0.964–1.168	0.228	0.288	19.8		
		TT *versus* CC	1.018	0.791–1.310	0.888	0.030	62.7		
ARMS-PCR	2	T *versus* C	0.984	0.613–1.578	0.947	0.545	0.0		
		TT + CT *versus* CC	0.906	0.494–1.661	0.749	0.596	0.0		
		TT *versus* CC + CT	1.431	0.368–5.567	0.605	—	—		
		CT *versus* TT	0.871	0.468–1.619	0.662	0.635	0.0		
		TT *versus* CC	1.379	0.338–5.636	0.654	—	—		
Sequenom MassArry	1	T *versus* C	0.761	0.462–1.254	0.284	—	—		
		TT + CT *versus* CC	0.611	0.327–1.140	0.121	—	—		
		TT *versus* CC + CT	1.184	0.446–3.144	0.735	—	—		
		CT *versus* TT	0.524	0.259–1.058	0.071	—	—		
		TT *versus* CC	0.954	0.351–2.589	0.926	—	—		
Sample source									
Peripheral blood	17	T *versus* C	0.936	0.847–1.035	0.195	0.000	70.6		
		TT + CT *versus* CC	0.927	0.823–1.043	0.208	0.000	61.8		
		TT *versus* CC + CT	0.898	0.731–1.102	0.303	0.000	67.9		
		CT *versus* TT	0.946	0.849–1.054	0.315	0.011	49.5		
		TT *versus* CC	0.866	0.687–1.093	0.227	0.000	71.9		
Paraffinized tissue	2	T *versus* C	0.927	0.632–1.360	0.698	0.500	0.0		
		TT + CT *versus* CC	0.784	0.476–1.291	0.339	0.333	0.0		
		TT *versus* CC + CT	1.606	0.568–4.546	0.372	0.795	0.0		
		CT *versus* TT	0.713	0.395–1.284	0.260	0.255	22.8		
		TT *versus* CC	1.400	0.479–4.090	0.539	0.975	68.6		

Subgroup analysis (Supplementary Appendix S2) was performed to evaluate the heterogeneity among the studies based on race, genotyping method, and sample type. According to our subgroup analysis, heterogeneity was significantly reduced among all the race subgroups. In the Asian population, a decreased risk of prostate cancer was associated with the C677T polymorphism (T allele vs. C allele: OR = 0.759, 95% CI 0.669–0.861, *p* < 0.001, I^2^ = 28.1%, *p*_H_ = 0.249; TT + CT vs. CC: OR = 0.720, 95% CI 0.638–0.812, *p* < 0.001, I^2^ = 0.0%, *p*_H_ = 0.409; TT vs. CC + CT: OR = 0.719, 95% CI 0.617–0.838, *p* < 0.001, I^2^ = 0.0%, *p*_H_ = 0.442; CT vs. CC: OR = 0.761, 95% CI 0.670–0.865, *p* < 0.001, I^2^ = 0.0%, *p*_H_ = 0.416; TT vs. CC: OR = 0.620, 95% CI 0.522–0.737, *p*=<0.001, I^2^ = 0.0%, *p*_H_ = 0.477). With respect to the different genotyping methods, heterogeneity in the dominant and allelic models was reduced in the TaqMan and ARMan-ARMS-PCR groups. The overdominant model showed reduced heterogeneity in the PCR-RFLP, TaqMan, and ARMan-ARMS-PCR groups. However, the heterogeneity remained relatively high in the other models. In the PCR-RFLP group, a decreased risk of prostate cancer was observed in the comparison of the dominant and overdominant models (TT + CT vs. CC: OR = 0.839, 95% CI 0.720–0.977, *p* = 0.024, I^2^ = 41.8%, *p*_H_ = 0.070; CT vs. CC: OR = 0.848, 95% CI 0.746–0.965, *p* = 0.012, I^2^ = 18.7%, *p*_H_ = 0.266). In the subgroup analysis based on sample type, even after excluding two studies that extracted genes from paraffin-embedded tissues (Singal and Vidal), heterogeneity remained high. However, in the paraffinized tissue subgroup, heterogeneity significantly decreased in all models except for the additive model. No significant correlation was found in any of the genetic models (Table 2).

### 3.3 Association between the A1298C (rs1801131) polymorphism and prostate cancer susceptibility

**FIGURE 3:**
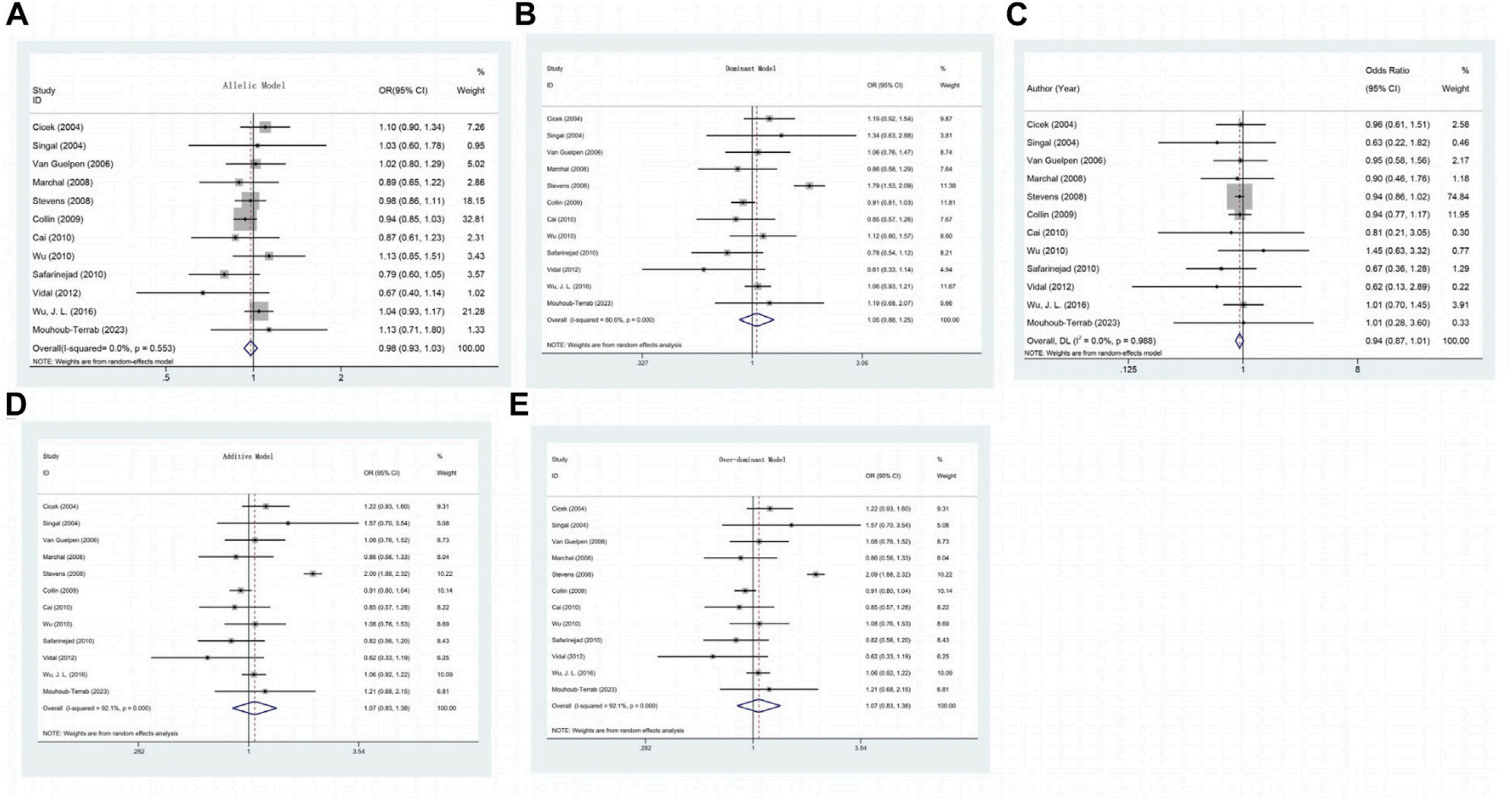
**(A)** Forest plot of the association between MTHFR gene A1298C polymorphism and PCa risk under allelic model. **(B)** Forest plot of the association between MTHFR gene A1298C polymorphism and PCa risk under dominant model. **(C)** Forest plot of the association between MTHFR gene A1298C polymorphism and PCa risk under recessive model. **(D)** Forest plot of the association between MTHFR gene A1298C polymorphism and PCa risk under over-dominant model. **(E)** Forest plot of the association between MTHFR gene A1298C polymorphism and PCa risk under additive model.

The association between the A1298C (rs1801131) polymorphism and prostate cancer risk was not found to be significant in any of the genetic models (Table 2) (C allele vs. A allele: OR = 0.979, 95% CI 0.928–1.032, *p*_H_ = 0.553, I^2^ = 0.0%, *p* = 0.427; CC + AC vs. AA: OR = 1.046, 95% CI 0.876–1.251, *p*_H_ < 0.001, I^2^ = 80.6%, *p* = 0.618; CC vs. AA + AC: OR = 0.939, 95% CI 0.874–1.010, *p*_H_ = 0.988, I^2^ = 0.0%, *p* = 0.091; AC vs. AA: OR = 1.071, 95% CI 0.828–1.383, *p*_H_ < 0.001, I^2^ = 92.1%, *p* = 0.602; CC vs. AA: OR = 0.933, 95% CI 0.803–1.083, *p*_H_ = 0.938, I^2^ = 0.0%, *p* = 0.362) (Table 3) (Figures 3A–E).

**TABLE 3:** Meta-analysis between MTHFR A1298C polymorphism and PCa risk under genetic models.

Classification	N	Contrasting model	OR	95%CI	P	Heterogeneity		Publication bias	
						P Of Q-test	I^2^%	P Of Begg test	P Of Egger test
Total	19	C *versus* A	0.979	0.928–1.032	0.427	0.553	0.0	0.451	0.697
		CC + AC *versus* AA	1.046	0.876–1.251	0.618	0.000	80.6	0.945	0.634
		CC *versus* AA+ AC	0.939	0.874–1.010	0.091	0.988	0.0	0.451	0.547
		AC *versus* CC	1.071	0.828–1.383	0.602	0.000	92.1	0.537	0.188
		CC *versus* AA	0.933	0.803–1.083	0.362	0.938	0.0	0.244	0.052
Race									
Caucasian	6	CC + AC *versus* AA	0.908	0.820–1.005	0.063	0.528	0.0		
		AC *versus* CC	0.915	0.823–1.019	0.106	0.629	0.0		
Asian	3	CC + AC *versus* AA	1.045	0.928–1.177	0.469	0.535	0.0		
		AC *versus* CC	1.043	0.923–1,179	0.501	0.594	0.0		
Mixed	3	CC + AC *versus* AA	1.464	1.052–2.037	0.024	0.029	71.7		
		AC *versus* CC	1.615	1.037–2.514	0.034	0.001	85.1		
Genotyping method									
PCR-RFLP	6	CC + AC *versus* AA	1.045	0.937–1.164	0.430	0.393	3.6		
		AC *versus* CC	1.056	0.949–1.175	0.316	0.449	0.0		
TaqMan	5	CC + AC *versus* AA	1.130	0.785–1.626	0.511	0.000	91.4		
		AC *versus* CC	1.176	0.724–1.909	0.513	0.000	96.3		
Sequenom MassArry	1	CC + AC *versus* AA	0.611	0.327–1.140	0.121	—	—		
		AC *versus* CC	0.624	0.326–1.191	0.153	—	—		
Sample source									
Peripheral blood	11	CC + AC *versus* AA	1.035	0.862–1.244	0.710	0.000	82.3		
		AC *versus* CC	1.048	0.805–1.366	0.726	0.000	92.8		
Paraffinized tissue	1	CC + AC *versus* AA	1.345	0.628–2.880	0.446	—	—		
		AC *versus* CC	1.570	0.696–3.540	0.277	—	—		

Furthermore, in the models with high heterogeneity, subgroup analysis (Supplementary Appendix S2) based on race, genotyping method, and sample type did not reveal any significant associations. However, it is worth noting that the studies by Cicek, Singal, and Stevens included multiethnic populations in the United States, and the subgroup analysis indicated an increased risk of prostate cancer in the comparison of the dominant and overdominant models (CC + AC vs. AA: OR = 1.464, 95% CI 1.052–2.037, *p* = 0.024, I^2^ = 71.7%, *p*_H_ = 0.029; AC vs. AA: OR = 1.615, 95% CI 1.037–2.514, *p* = 0.034, I^2^ = 85.1%, *p*_H_ = 0.001). For the dominant and overdominant models, a significant reduction in heterogeneity was observed among Caucasians and Asians. In studies using different genotyping methods, heterogeneity was significantly reduced compared with that in those utilizing PCR-RFLP. According to the subgroup analysis based on sample type, heterogeneity remained high even after excluding two studies (Singal) that extracted genes from paraffin-embedded tissues (Table 3).

### 3.4 Sensitivity analysis and publication bias

After systematically excluding each individual study and conducting a second meta-analysis to pool effect sizes, it was found that the analysis results for all models regarding C677T remained consistent with the primary analysis, demonstrating the robustness of the main findings (Porta et al., 2014). However, during the sensitivity analysis of A1298C, a notable alteration was observed in the homozygous (CC vs. AA) model comparison when the study by Stevens was excluded due to its high sensitivity.

We employed both Begg’s and Egger’s tests to assess publication bias, and all studies demonstrated publication bias within an acceptable range (*p-value* > 0.05) (Supplementary Appendix S3).

## 4 Statistical analysis summary

This study investigates the relationship between MTHFR gene polymorphisms (C677T and A1298C) and genetic susceptibility to prostate cancer through a meta-analysis. Independent researchers searched databases like PubMed, Embase, Cochrane, and Web of Science until 19 December 2023, analyzing the association between MTHFR polymorphisms and prostate cancer risk using various genetic models. The meta-analysis included 26 case-control studies, involving 12,455 cases and 13,900 controls for C677T polymorphism, and 6,396 cases and 8,913 controls for A1298C polymorphism. Overall, no significant association was found between MTHFR polymorphisms and prostate cancer risk. However, in Asian populations, C677T polymorphism was associated with a reduced risk of prostate cancer, while in mixed-race groups in the USA, A1298C polymorphism was linked to an increased risk.

## 5 Discussion

In this meta-analysis, we evaluated the relationship between two SNPs (C677T and A1298C) in the methylenetetrahydrofolate reductase gene and prostate cancer risk based on 26 published studies. Overall, our analysis did not find evidence to suggest an association between the C677T or A1298C polymorphisms and the risk of prostate cancer in any genetic model. However, in subgroup analysis, we found that the C677T polymorphism may be associated with a reduced risk of prostate cancer in Asian populations, while the A1298C polymorphism may be associated with an increased risk in the American population.

The present analysis revealed that the C677T polymorphism is associated with a reduced risk of prostate cancer according to various genetic models, including the allele, dominant, recessive, and additive models. Li et al. (2012) also proposed a protective effect of the C677T allele polymorphism (Li and Xu, 2012). However, Chen et al. (2015) reported that the C677T polymorphism is associated with an increased risk of prostate cancer in East Asian populations, which contradicts previous findings (Chen et al., 2015). Moreover, Colin et al. (2009) and Zhang et al. (2012) did not find any association between the C677T polymorphism and the risk of prostate cancer. On the one hand, the present study incorporated the latest research and included a larger number of cases and controls, which enhances the reliability and statistical significance of the results (Collin et al., 2009; Zhang et al., 2012). On the other hand, the exclusion of studies with controls that deviated from Hardy-Weinberg equilibrium (HWE) might account for the divergent conclusions. HWE is an important principle in genetics that describes a stable state where genotype frequencies remain constant. A deviation of the control group from HWE may affect the accuracy of the study’s conclusions.

Regarding the A1298C polymorphism, previous meta-analyses showed that there is no association between this polymorphism and the risk of prostate cancer, regardless of whether the analysis was performed on the overall data or based on different regions, populations, genotyping methods, or prostate cancer stages (Li et al., 2012; Li and Xu, 2012). In our study, a subgroup analysis revealed increased susceptibility to prostate cancer in the mixed-race group when comparing the dominant and overdominant models. It is worth noting that the mixed-race group comprised individuals from various ethnic backgrounds, all of whom happen to be from the United States. Therefore, the correlation between the A1298C polymorphism and prostate cancer risk may be influenced by certain regional factors, such as dietary habits, climate conditions, and even cultural and socioeconomic factors.

Significant heterogeneity was observed in the association between the C677T and A1298C polymorphisms and prostate cancer risk. Therefore, subgroup analyses were performed based on race, genotyping method, and sample type. The heterogeneity was significantly reduced in different race groups, and a reduced risk of prostate cancer associated with the C677T polymorphism was observed in the Asian population. Furthermore, studies using the PCR-RFLP technique revealed that the C677T polymorphism decreased susceptibility to prostate cancer. These findings emphasize the importance of using consistent genotyping methods for accurate assessment of the association between the C677T polymorphism and prostate cancer risk. According to the subgroup analysis of the association between the A1298C polymorphism and prostate cancer risk, no significant associations were found. It is worth noting that while the studies by Cicek, Singal, and Stevens included a multiethnic population from the United States, which may consist of Caucasian, Asian, and black individuals, the analysis indicated an increased risk of prostate cancer in the comparison of the dominant and overdominant models. This finding suggested that the association between the A1298C polymorphism and prostate cancer susceptibility may be influenced by regional factors such as diet and requires further investigation.

In the race-based subgroup analysis for C667T and A1298C, a notable reduction in heterogeneity was observed, clearly highlighting the role of race as a factor contributing to heterogeneity in various studies. This underscores the importance of performing race-specific subgroup analyses in such research. According to the genotyping method-based subgroup analysis for C667T, the dominant and allelic models showed less heterogeneity in the TaqMan and ARMan-ARMS-PCR groups, while the overdominant model demonstrated reduced heterogeneity across the PCR-RFLP, TaqMan, and ARMan-ARMS-PCR groups. Similarly, for A1289C, studies utilizing the PCR-RFLP method displayed a marked decrease in heterogeneity. These observations indicate that the choice of genotyping method significantly influences heterogeneity, making it a crucial consideration in genetic research. Additionally, recognizing and accounting for methodological differences is vital when comparing or integrating study outcomes. According to our subgroup analysis focused on the sample source for C667T, all the models, except for the additive model, showed significantly less heterogeneity in the paraffin-embedded tissue subgroup. These findings suggest that the choice of sample source can contribute to heterogeneity and imply that paraffin-embedded tissues may be more consistent and reliable as a sample source in genetic studies.

Our meta-analysis is subject to several limitations. First, despite our efforts to incorporate the latest research, the number of included studies remained relatively small. Second, due to insufficient data availability, we were unable to adjust our results for other factors, such as patient age, sex, and environmental variables. Third, this meta-analysis exclusively examined the polymorphisms of two loci within Caucasian and Asian populations, thus precluding the assessment of the relationship between the C667T and A1298C polymorphisms and prostate cancer in other ethnic groups. Moreover, we must acknowledge the potential presence of publication bias since our analysis encompasses only published studies, and statistically nonsignificant results are often less likely to be published. Finally, the observed heterogeneity among the included studies may be attributed to various factors, including geographic distribution, participant demographics, study design, and methodological disparities.

## 6 Conclusion

In conclusion, our study provides an updated meta-analysis estimating the association between MTHFR gene polymorphisms and prostate cancer risk, incorporating a larger sample size than did previous studies. The C677T polymorphism may be associated with a reduced risk of prostate cancer in Asian populations, while the presence of the A1298C polymorphism may be associated with an increased risk in the U.S. population. Future studies should focus on large-scale, well-designed research incorporating regional factors such as diet and climate to confirm the association between MTHFR polymorphisms and prostate cancer susceptibility.
