# Supplementary material for: Associations between MTHFR gene polymorphisms (C677T and A1298C) and genetic susceptibility to prostate cancer: a systematic review and meta-analysis
Source: Front Genet. 2024 Jan 26;15:1343687. doi: 10.3389/fgene.2024.1343687 (PMC10853331; doi:10.3389/fgene.2024.1343687)
Supplement: Supplementary file 1 [file Table1.DOCX]

# Appendix 1. Search strategy

# Search strategy of PubMed

| #1 | "Prostatic Neoplasms"[MeSH Terms] OR "prostate neoplasms"[Title/Abstract] OR "prostate cancer"[Title/Abstract] OR "prostate tumor"[Title/Abstract] OR "prostate tumour"[Title/Abstract] OR "prostate carcinoma"[Title/Abstract] OR "prostatic neoplasms"[Title/Abstract] OR "prostatic cancer"[Title/Abstract] OR "prostatic tumor"[Title/Abstract] OR "prostatic tumour"[Title/Abstract] OR "prostatic carcinoma"[Title/Abstract] |
| --- | --- |
| #2 | "Methylene Tetrahydrofolate reductase"[Title/Abstract] OR "Methylenetetrahydrofolate reductase"[Title/Abstract] OR "Methylene-THF reductase"[Title/Abstract] OR "MTHFR"[Title/Abstract] |
| #3 | rs1801133"[All Fields] OR "Ala222Val"[All Fields] OR "C677T"[All Fields] OR "rs1801131"[All Fields] OR "Glu429Ala"[All Fields] OR "A1298C"[All Fields] |
| #4 | #1 AND #2 AND #3 |

Search strategy of Cochrane

| #1 | MeSH descriptor: [Prostatic Neoplasms] explode all trees |
| --- | --- |
| #2 | (Prostate Neoplasms):ti,ab,kw OR (Prostate cancer):ti,ab,kw OR (Prostate tumor):ti,ab,kw OR (Prostate tumour):ti,ab,kw OR (Prostate carcinoma):ti,ab,kw |
| #3 | (Prostatic Neoplasms):ti,ab,kw OR (Prostatic cancer):ti,ab,kw OR (Prostatic tumor):ti,ab,kw OR (Prostatic tumour):ti,ab,kw OR (Prostatic carcinoma):ti,ab,kw |
| #4 | #1 OR #2 OR #3 |
| #5 | (Methylene Tetrahydrofolate Reductase):ti,ab,kw OR (Methylenetetrahydrofolate reductase):ti,ab,kw OR (Methylene-THF reductase):ti,ab,kw OR (MTHFR):ti,ab,kw |
| #6 | (rs1801133) OR (Ala222Val) OR (C677T) |
| #7 | (rs1801131) OR (Glu429Ala) OR (A1298C) |
| #8 | #6 OR #7 |
| #9 | #4 AND #5 AND #8 |

Search strategy of Cochrane

| #1 | 'prostate tumor'/exp |
| --- | --- |
| #2 | 'prostate cancer':ti,ab,kw OR 'prostate tumor':ti,ab,kw OR 'prostate tumour':ti,ab,kw OR 'prostate carcinoma':ti,ab,kw OR 'prostate neoplasms':ti,ab,kw OR 'prostatic neoplasms':ti,ab,kw OR 'prostatic cancer':ti,ab,kw OR 'prostatic tumor':ab,ti OR 'prostatic tumour':ti,ab,kw OR 'prostatic carcinoma':ti,ab,kw |
| #3 | #1 OR #2 |
| #4 | 'methylene tetrahydrofolate reductase':ti,ab,kw OR 'methylenetetrahydrofolate reductase':ab,ti OR 'methylene-thf reductase':ti,ab,kw OR mthfr:ti,ab,kw |
| #5 | rs1801133 OR ala222val OR c677t OR rs1801131 OR glu429ala OR a1298c |
| #6 | #3 AND #4 AND #5 |

Search strategy of Web of Science

| #1 | Prostate Neoplasms (Topic) OR Prostate cancer (Topic) OR Prostate tumor (Topic) OR Prostate tumour (Topic) OR Prostate carcinoma (Topic) OR Prostatic Neoplasms (Topic) OR Prostatic cancer (Topic) OR Prostatic tumor (Topic) OR Prostatic tumour (Topic) OR Prostatic carcinoma (Topic) |
| --- | --- |
| #2 | Methylene Tetrahydrofolate Reductase (Topic) OR Methylenetetrahydrofolate reductase (Topic) OR Methylene-THF reductase (Topic) OR MTHFR (Topic) |
| #3 | rs1801133 (All Fields) OR Ala222Val (All Fields) OR Ala222Val (All Fields) OR rs1801131 (All Fields) OR Glu429Ala (All Fields) OR A1298C (All Fields) |
| #4 | ' #1 AND #2 AND #3 |
