# Supplementary material for: Associations between MTHFR gene polymorphisms (C677T and A1298C) and genetic susceptibility to prostate cancer: a systematic review and meta-analysis
Source: Front Genet. 2024 Jan 26;15:1343687. doi: 10.3389/fgene.2024.1343687 (PMC10853331; doi:10.3389/fgene.2024.1343687)

**Appendix 2. subgroup analysis**

**Subgroup analysis of A1298C dominant model**


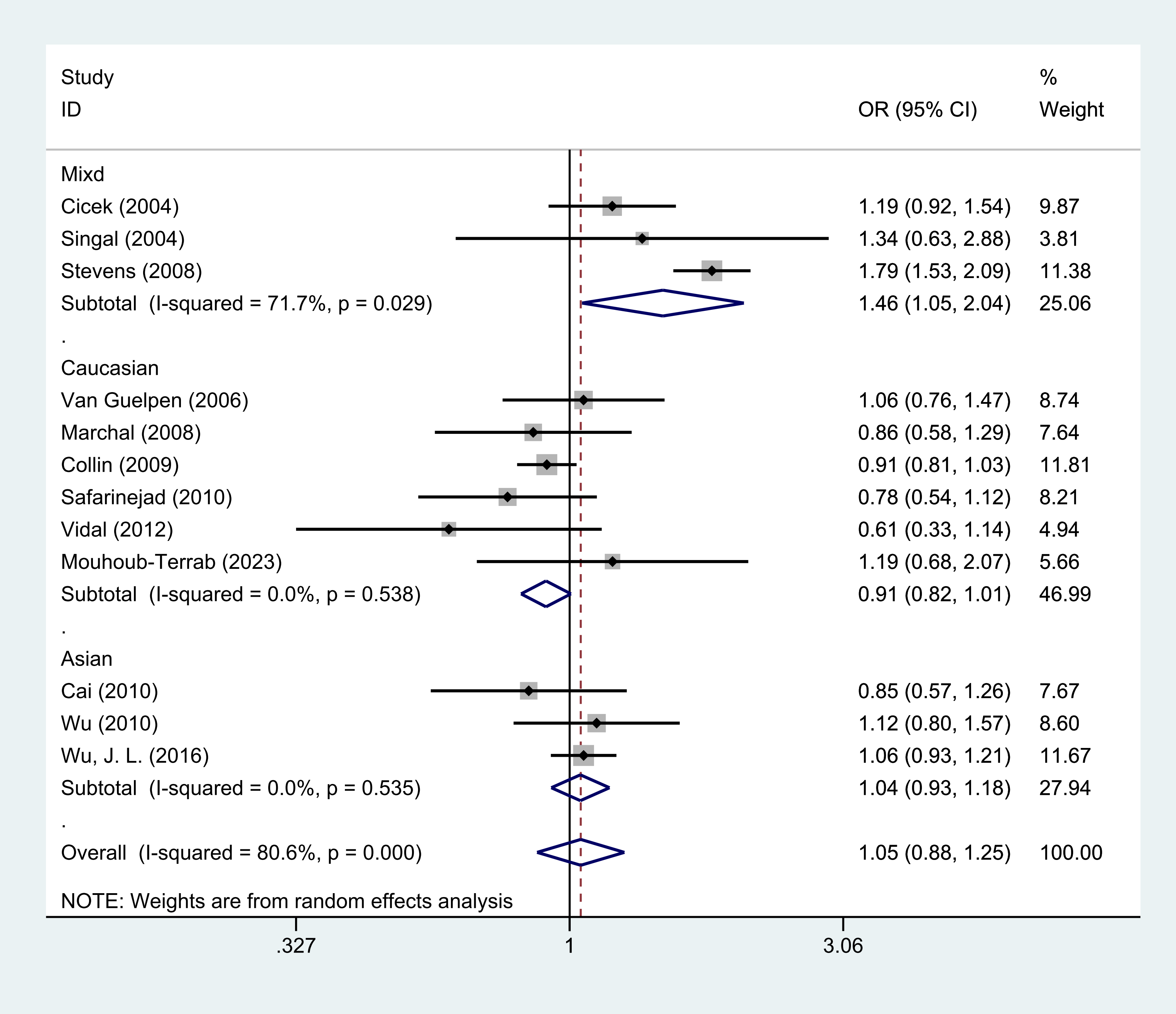


**Subgroup analysis of A1298C over-dominant model**


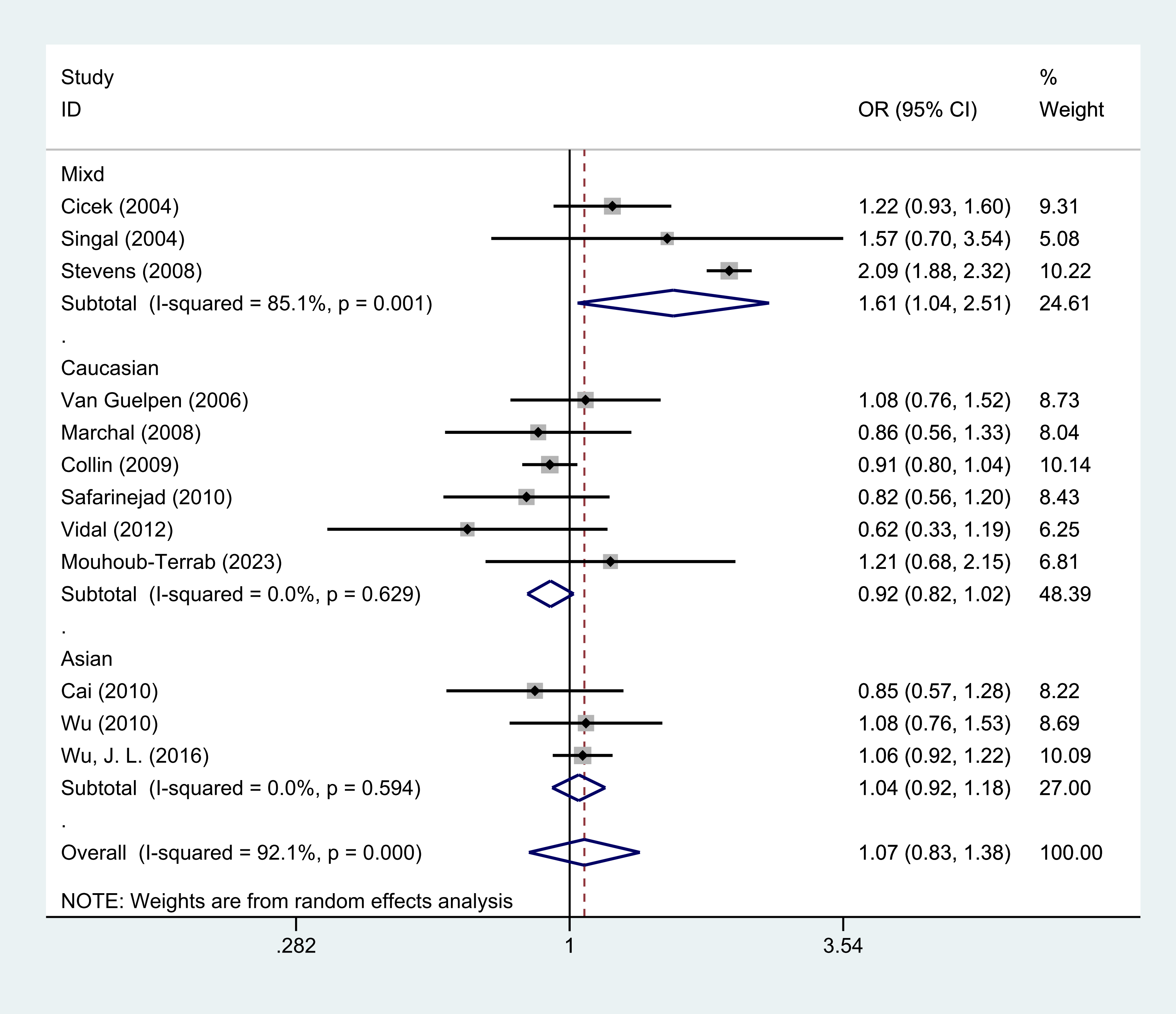


**Subgroup analysis of C677T additive model**





**Subgroup analysis of C677T Allelic model**





**Subgroup analysis of C677T Dominant model**





**Subgroup analysis of C677T recessive model**





**Subgroup analysis of C677T over-dominant model**


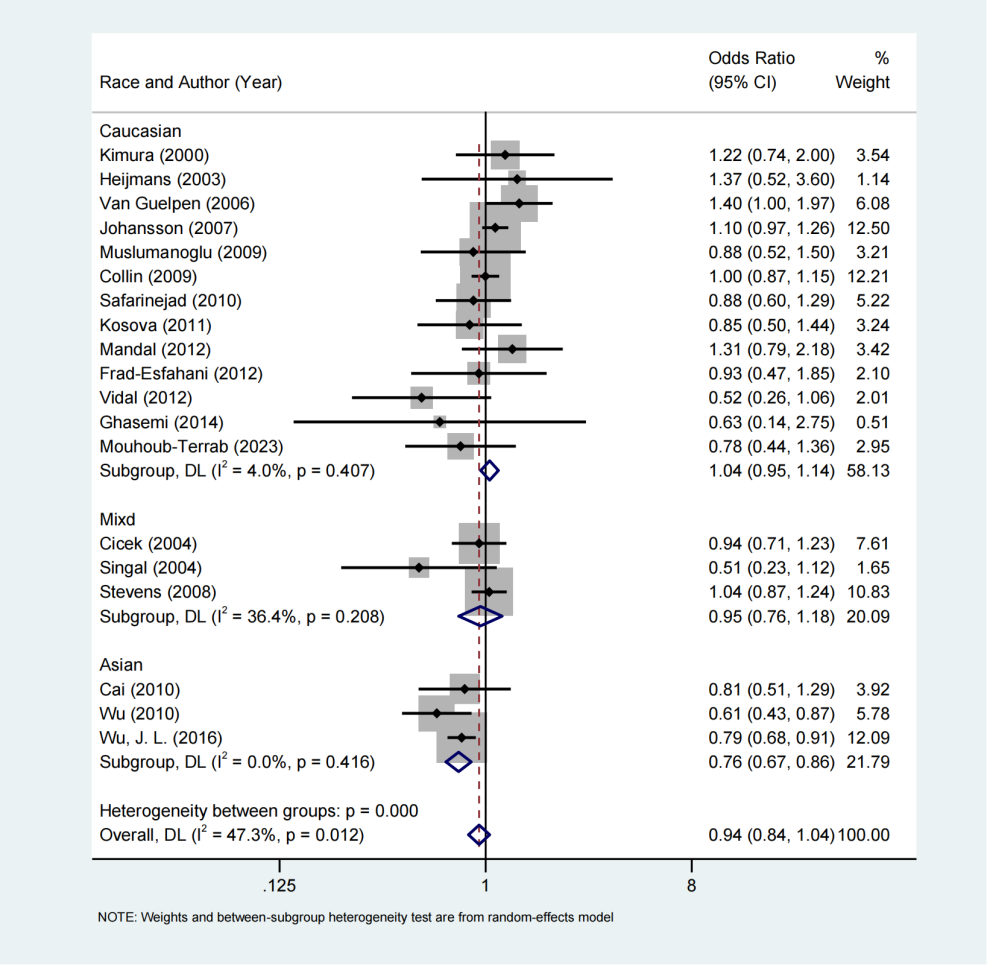

Supplement: Supplementary file 2 [file Table2.DOCX]
