# Supplementary material for: Associations between MTHFR gene polymorphisms (C677T and A1298C) and genetic susceptibility to prostate cancer: a systematic review and meta-analysis
Source: Front Genet. 2024 Jan 26;15:1343687. doi: 10.3389/fgene.2024.1343687 (PMC10853331; doi:10.3389/fgene.2024.1343687)
Supplement: Supplementary file 3 [file Table3.DOCX]

**Appendix 3. Sensitivity and publication bias**

**C677T Allelic model**

P=0.780

P=0.775

**C677T Dominant model**

P=0.294

P=0.508

**C677T recessive model**

P=0.289

P=0.837

**C677T over-dominant model**

P=0.234

**C677T recessive model**

P=0.289

**A1298C allelic model**

P=0.451

P=0.697

**A1298C dominant model**

P=0.945

P=0.634

**A1298C recessive model**

P=0.451

P=0.547

**A1298C over-dominant model**

P=0.537

P=0.188

**A1298C additive model**


P=0.244

P=0.052
